# Living in existential exile: women’s lived experience of intimate partner violence during the breastfeeding period

**DOI:** 10.1080/17482631.2025.2507313

**Published:** 2025-05-20

**Authors:** Ida Gustafsson, Aleksandra Jarling, Katarina Karlsson, Lina Palmér

**Affiliations:** a Faculty of Caring Science, Work Life and Social Welfare, University of Borås, Borås, Sweden; b PreHospen Centre for Prehospital Research, University of Borås, Borås, Sweden

**Keywords:** Breastfeeding, caring, lived experience, intimate partner violence (IPV), lifeworld hermeneutics, qualitative research, women

## Abstract

**Purpose:**

Intimate partner violence is a global health issue with physical, mental, and existential impacts. It affects women throughout their lives, including the breastfeeding period. Gaining an understanding of existential dimensions could potentially inspire individualized, health-oriented care. This study aims to explain and understand women’s lived experience of intimate partner violence during the breastfeeding period.

**Methods:**

A lifeworld hermeneutic approach guided the interpretative analysis of nine lifeworld interviews and forty-nine written lifeworld stories of women exposed to intimate partner violence during breastfeeding.

**Results:**

The interpretations show that intimate partner violence during the breastfeeding period means to breastfeed under attack in an objectified and provocative female body while feeling abandoned and entrapped in an incomprehensible reality. The interpretations are abstracted into a main interpretation: being forced into an existential exile that entails an ambiguous passive-active resistance.

**Conclusions:**

Exposure to intimate partner violence during breastfeeding is a forced existential exile in a vulnerable situation. Women are forced into unauthentic lives, where their whole being is questioned, and active resistance is inhibited by limited freedom. Awareness of the lived experience of IPV during breastfeeding is essential for healthcare professionals to help reduce the suffering and enhance the health and well-being of women exposed to IPV.

## Introduction

Intimate Partner Violence (IPV) affects one out of every three women globally at some point in their lives (World Health Organization WHO, 2021). Reported prevalence during childbearing varies across countries (Daoud et al., 2017; Katole et al., 2023; Sánchez et al., 2023; Velonis et al., 2017). Study differences in methods, definitions, and participant groups make comparisons difficult, but IPV is clearly common during this period (Barnes et al., 2024; Bowen et al., 2005; Daoud et al., 2012). In Sweden, around 2.5% of women experience IPV during pregnancy and 3.3% experience IPV postpartum (Finnbogadóttir & Dykes, 2016). Childbearing, including breastfeeding, is a vulnerable period in relation to IPV because the women no longer have only themselves to protect (Håland, 2014). IPV may be *physical*, *psychological, sexual, material, social, economic*, and/or *digital*; including threats, manipulation, rape, property destruction, isolation, or coercion to reveal security codes for bank cards and social media accounts (United Nations UN, n.d.). IPV during the breastfeeding period has been shown to manifest as a continuum of violence within a patriarchal context, incorporating intertwined experiences of being *accused, devalued, neglected, controlled, opposed, forced to adapt*, and *punished*. This includes being prevented from breastfeeding or making independent breastfeeding choices, being belittled as a breastfeeding mother, and being coerced into sex, while breastfeeding (Gustafsson et al., 2024). Help-seeking may be prevented by factors such as isolation, inability to identify as exposed to IPV, fear of consequences, lack of resources, stigma, self-blame, and distrust of support agencies (Robinson et al., 2020). Thus, IPV and other types of violence against women are global health issues that impede the health and well-being of women and children, sustainable development, and human rights (World Health Organization WHO, 2024). Breastfeeding has the potential to reduce health problems for both women and children (Global Breastfeeding Collective, 2017) and contributes to achieving the Sustainable Development Goals of the 2030 Agenda (United Nations UN, 2015), but IPV could negatively impact breastfeeding (Mezzavilla et al., 2018; Normann et al., 2020).

Existential issues are always present in human life, especially during periods of struggle and suffering (Palmér et al., 2023), making them relevant to study in the context of IPV during breastfeeding. Existential issues concern questions of being and human “meaning-making” in relation to life and life events (Palmér et al., 2023). It highlights the conditions of existence, such as the finiteness of life and the concurrent freedom and “un-freedom” of being cast into life under specific circumstances, yet free and responsible for one’s choices (Aho, 2020). Breastfeeding has been described as an existential issue and a meaningful act in previous lifeworld theoretical research. Breastfeeding difficulties may evoke existential concerns about being lost and not finding one’s way into motherhood (Palmér, 2015), which could lead to experiences of guilt, shame, and a sense of failure as a mother (Morns et al., 2021; Palmér, 2015). Exposure to IPV during childbearing has also been shown to be an existential experience (Engnes et al., 2012). An existential focus in research visualizes phenomena such as exposedness and suffering (Palmér et al., 2023) through the investigation of lived experience. Lived experience originates from the unreflected, natural attitude of the lifeworld, which is unique to every person and is based on our natural ability to make experiences meaningful by relating them to previous experiences (Dahlberg & Dahlberg, 2020). Lifeworld-based research urges healthcare professionals to heed existential issues and concerns and base their caring on human experience to reduce suffering and enhance health and well-being (Palmér et al., 2023). This study aims to explain and understand women’s lived experience of IPV during the breastfeeding period.

## Materials and methods

The phenomenon *IPV during the breastfeeding period* has been studied using a lifeworld hermeneutic approach based on Reflective Lifeworld Research (Dahlberg et al., 2008; Palmér et al., 2023) and ontologically and epistemologically rooted in the philosophies of Husserl (Husserl, 1970, 1990), Heidegger (Heidegger, 1998), Gadamer (Gadamer, 1997), and Ricoeur (Ricoeur, 1976, 1998). The lifeworld hermeneutic approach is meaning-oriented and takes its starting point in the lifeworld (Dahlberg et al., 2008; Palmér et al., 2023). According to Husserl’s Lifeworld theory, human understanding is characterized by a natural attitude, an individual, pre-reflected, and taken-for-granted approach to everyday life in which our pre-understanding forms our understanding of something as something. We may share our world with other people to some extent, but we can never entirely understand another lifeworld. The natural attitude is intentional and thus always directed towards something. To truly understand, the researcher needs to shift from a natural attitude to a more reflective one to allow the phenomenon to “reveal itself” in a phenomenological sense (Husserl, 1970, 1990). The methodological principles guiding researchers in examining lived experience are *openness* to new and surprising aspects in data, *pliability* towards the phenomenon, and *bridling* of both pre-understanding and new phenomena understanding (Dahlberg et al., 2008; Palmér et al., 2023). However, Heidegger highlights the difficulties of completely abandoning the natural attitude by claiming that understanding is naturally based on interpretation (Heidegger, 1998), while Gadamer emphasizes the awareness that pre-understanding influences our “horizons” of understanding, making it more challenging to comprehend phenomena in novel ways (Gadamer, 1997). Ricoeur argues that the interpretative process is dialectical and cumulative, resulting in an explanation and understanding of the phenomenon (Ricoeur, 1976). The explanations should be intentional, shedding light on how and why we understand instead of focusing on cause and effect (Ricoeur, 1998). This view has strongly influenced the lifeworld hermeneutic approach (Dahlberg et al., 2008; Palmér et al., 2023).

### Data collection and participants

Data were collected in 2022 and 2023 and consist of written lifeworld stories and lifeworld interviews of women with lived experience of IPV during the breastfeeding period. The breastfeeding period was defined as the time during which they breastfed or wanted to breastfeed to some degree. A lifeworld story or interview focuses on phenomena meaning, as experienced by participants, and starts with an open-ended question (Dahlberg et al., 2008; Palmér et al., 2023). The participants were found through Swedish social media groups using advertisements approved by the group administrator. To capture phenomenon variation while acknowledging women’s tendency not to define themselves as IPV victims (Crann & Barata, 2015), the term “IPV” was avoided in the advertisement in favour of “broader” expressions like: “Have you experienced exposedness and/or lack of support in your partner relationship during the breastfeeding period?”.

#### Written lifeworld stories

The advertisements were linked to a questionnaire in a secure, web-based platform, where written information about study participation and contact information to the corresponding author was distributed. In accordance with the methodological principles, the information clearly indicated the authors’ openness to participants’ lived experience of the phenomenon. However, to encourage phenomenon-focused, meaningful, and varied narratives in the absence of follow-up questions, questionnaire participants were given general examples of what exposedness could entail, based on previous IPV research (e.g., *“This could involve feeling that your breastfeeding decisions were not respected, that your self-confidence was negatively affected, or that your partner exhibited jealousy or anger towards you and the child”*). Forty-nine women participated in writing via the questionnaire, which began with background questions, followed by an invitation to freely write their lifeworld stories in response to the open-ended question: *Would you please tell me about your experience of exposedness in a partner relationship during your breastfeeding period?* The written lifeworld stories consist of approximately 9,000 words in total.

#### Lifeworld interviews

Those unwilling to participate in writing or wishing to elaborate on their stories could participate through an interview after contacting the corresponding author via email. Thirteen women reached out, but four did not respond to the first author’s (IG) attempts to schedule an interview. Eventually, nine women were interviewed by IG. Owing to the national recruitment of study participants and the aftermath of the COVID-19 pandemic, interviews were conducted via encrypted video conferencing (seven) or by telephone (two). Because of anonymization, it is unknown whether the interviewees also participated in writing. The interviews began with the same open-ended question as the written lifeworld stories. Follow-up questions, such as *Can you elaborate on that?* or *How did that make you feel?* were used to clarify and deepen the descriptions. The audio-recorded interviews lasted for about 60–120 minutes and were transcribed verbatim.

#### Participants

All participants experienced IPV in a heterosexual relationship that lasted 1–10 years, and all but two were born in Sweden. Some remained in the relationship, while others were separated. They were 25–45 years old and had a minimum of 3 years of upper secondary school and a maximum of >3 years post-secondary education. They had breastfed (or wanted to breastfeed) one to four children. In relation to the group that participated in writing, the interview group was older, better educated, and had more often separated from their partner. At the group level, the participants described experiences of physical, mental, sexual, economic, material, social, and digital IPV, but the types, frequency, and manifestations of IPV varied individually.

### Data analysis

Lifeworld hermeneutic methodological principles were used during the analysis to achieve study validity (Dahlberg et al., 2008; Palmér et al., 2023). Lifeworld hermeneutics makes no claim of holding the one and only truth, but it is not a random guess (Dahlberg et al., 2008). Interpretations must be (the most) meaningful in relation to the phenomenon and aim (Palmér et al., 2023). The authors were pliable to the phenomenon, aim, and data when choosing the research method. The approach is characterized by a constant movement between the whole and parts of the data, interpretations, and main interpretation to ensure data support and meaningfulness and avoid contradictions. This was done by the first author (IG) initially familiarizing with the data through repeated reading (the whole), followed by marking, analysing, and grouping meaning-units into interpretations (the parts). Tentative interpretations were validated by efforts to bridle pre-understanding and slow down the process of understanding through openness to surprising aspects in the data and continuous reflections within the research group. Finally, a main interpretation that unifies and deepens the interpretations and explains the phenomenon in an understandable way was developed (Dahlberg et al., 2008; Palmér et al., 2023). Interpretations are illustrated in the results with participant quotes from multiple written and verbal lifeworld stories. For clarity, insignificant words and repetitions were removed or rephrased and personal information was anonymized without altering the meaning.

### Ethical considerations

The Regional Ethical Review Board in Linköping, Sweden approved the study (ref. no. 2022 -01397-01). In compliance with the Declaration of Helsinki (World Medical Association [WMA], 2024), all participants were informed in writing about the purpose of the study, what participation would entail for them, how their data would be protected and used, and their right to withdraw at any time without consequences. Regarding the written lifeworld stories via the web-based questionnaire, consent was deemed obtained upon form submission. Women participating in lifeworld interviews received verbal study information and the opportunity to pose questions before the interview. Their informed consent was obtained verbally and documented by the interviewer, as approved by the Regional Ethical Review Board.

In accordance with WHO’s ethical and safety recommendations on violence against women (WHO, 2001, 2016), participant safety and confidentiality were paramount. Participants could choose their mode of participation (writing or interview) and where not required to disclose their location. Personal data collection was minimized by gathering background data at a group level and allowing the use of pseudonyms. Information about support organizations was included in the web-based questionnaire and the interviewer remained after the interviews to provide participants with the opportunity to express any requirements for further care or support. The interviewer also had an awareness that the interviews could become emotional. Participants were encouraged to take breaks and, if needed, continue the interview on another occasion. However, they expressed both confidence in managing their emotions and the need to narrate their experiences without interruption. One participant requested two interview sessions for practical reasons.

## Results

IPV during the breastfeeding period could be explained and understood through five interpretations and a unifying main interpretation. The interpretations are: *Breastfeeding under attack—a reason to give up or fight back; Breastfeeding in an objectified and provocative female body; Breastfeeding in abandonment; Breastfeeding in entrapment*, and *Breastfeeding in an incomprehensible reality*. The main interpretation shows that IPV during the breastfeeding period means *Being forced into an existential exile that entails an ambiguous passive-active resistance.*

### Breastfeeding under attack—a reason to give up or fight back

The experience of IPV during breastfeeding could be interpreted as breastfeeding under attack. Regardless of IPV type or manifestation, being attacked by one’s partner entails living in a fragile and stressful reality with experiences such as *drowning* or *breastfeeding on a sinking ship*, calling for constant crisis preparedness:

I was in a situation of… like when bombs fall. There’s a war going on.//So, I was in… a kind of high stress level, all the time…//It totally diminished the breastfeeding.

Regardless of whether the partner demands that the woman achieve unrealistic breastfeeding goals or cease breastfeeding altogether, such attacks mean being prevented from the possibility of relaxation and presence, potentially contributing to the deprioritisation and reduction of breastfeeding. Being robbed of the opportunity to be the breastfeeding mother imagined, being unable to put the child first, and/or being unable to protect him or her from IPV consequences, jeopardizes the joy of breastfeeding, as one woman describes:

It was not good conditions for breastfeeding//It was never calm//Breastfeeding just had to ‘slip into life’… It wasn’t priority.

Breastfeeding under attack also means being drawn into a power struggle, where refusing to comply with one’s partner sometimes results in being forced to negotiate oneself away. Being forced to abandon breastfeeding goals, adapting breastfeeding decisions to the partner’s wishes, or withholding decisions from him could reduce attacks. However, the child’s need to breastfeed and be cared for is perceived as non-negotiable, unlike the woman’s own needs. One woman describes how the attacks did not make her give up her breastfeeding, but rather pretend she did:

It was in some strange way that I had to focus on the baby’s needs, and at the same time pretend that I didn’t.

Sometimes, the attacks imply a source of strength to fight back and more overtly advocate for breastfeeding decisions and rights. One woman illustrates how the attacks unexpectedly awakened a competitive drive:

The more he questioned it [the breastfeeding], the more I stuck to it//It’s between her and me.

### Breastfeeding in an objectified and provocative female body

The experience of IPV during breastfeeding could also be interpreted as breastfeeding in an objectified and provocative female body. Living in a female body with the ability to independently carry, nourish, and comfort a child may highlight the partner’s bodily inability and provoke his perceived exclusive right to the woman’s body:

The father thought that this new baby came between me and him and was annoyed that she… should breastfeed all the time or have me and have my body.

Being objectified through devaluation of the female breastfeeding body implies a shift of power in favour of the partner. Objectification means being considered and treated as an object and being valued based on obedience and bodily performance. Having breastfeeding problems when the partner views breastfeeding as a female duty is deemed a failure and results in devaluation. Breastfeeding in an objectified and provocative female body sometimes “justifies” insults unthinkable in other contexts:

He said something that was so … very cruel to say. Both sexualising, degrading – and that he at the same time compared me to an animal. There were so many layers to the insult … that he intended as a joke, but it really wasn’t a funny joke at all.

Viewing one’s own body as a provider of child nutrition and safety, while simultaneously being viewed as a sexual object by one’s partner, implies no longer owning one’s own body or breasts and may lead to discomfort in breastfeeding and internalization of an objectifying view of the female body:

He had sex with me while I was breastfeeding…//I can absolutely think it’s humiliating today… But in that situation… I was probably okay with it being a good solution to make everyone happy at the same time.

### Breastfeeding in abandonment

The experience of IPV during breastfeeding could be interpreted as breastfeeding in abandonment. Breastfeeding means being vulnerable to abandonment because the responsibility for the child imposes an increased need for support. Being abandoned in this situation entails being forced to assume full parental responsibility and becoming the family’s pillar of support, due to the partner’s lack of interest or ability to be involved. However, being “the strong one” actually causes feelings of powerlessness and loneliness. Two women recount their experiences of complete abandonment:

[Breastfeeding] wasn’t something that had to do with… our parenting. But it was something that had to do with my parenting – whether I wanted to do it or not – because he didn’t care.

Before I left him [the abusive partner] … I wanted to die. But… I knew that I was the only one taking care of our child so I couldn’t.

Being abandoned in the vulnerable breastfeeding situation and having a partner that does not recognize the importance of, or effort involved in, breastfeeding could also result in being abandoned with breastfeeding problems and ill health, especially when breastfeeding against the partner’s will. Being abandoned means living with a counter-parent instead of a co-parent. One woman describes:

He always got hung up on the fact that he couldn’t help me and relieve me with breastfeeding and therefore was completely at a loss to be of any help whatsoever.

Being abandoned entails feeling unimportant and unloved, particularly if the IPV has led to isolation from family and friends. Experiencing that parts of you could *“die”* without anyone noticing or caring imposes a risk to become a *“shell”* of your former self. One woman states:

Me as a human being, me as a mother, me as a woman, I wasn’t important.

### Breastfeeding in entrapment

The experience of IPV during breastfeeding could also be interpreted as breastfeeding in entrapment. The unsharable nature of breastfeeding means being bound to the child, home, and partner. Becoming a new parent seems to reinforce the desire to be a “happy family”, increasing co-parent dependence. This means there is a risk of further entrapment if the partner takes advantage of the situation. Being left alone with the time-consuming task of breastfeeding or being hindered from leaving the home imposes a risk of isolation from care and support:

[Breastfeeding] was a way for him, in relation to other people, to [say]: “No, we can’t meet now, because she’s going to do this”.

Breastfeeding in entrapment also entails the inability to fully control and take responsibility for breastfeeding. Being prevented from breastfeeding involves punishment and entrapment in a situation of unableness to nurture a hungry child. Fearing a separation will force the child to spend time alone with the partner may reinforce the entrapment. Even separation from the partner does not guarantee escape, as mutual parental responsibilities may result in lifelong entrapment. One woman describes her post-separation experience as follows:

Even though we were breastfeeding, he would [demand to] have the baby for several hours a day. Alone//I came home and breastfed two hours into their visitation… And then I left again.

Previous IPV experience could result in ongoing entrapment. One woman illustrates how bodily memories of sexual IPV were trigged when she was compelled to breastfeed in front of her ex-partner, as the authorities deemed him entitled to see the child, but the child too young to meet him without the mother:

Just seeing him started such incredible… physical reactions//To have to expose something so intimate. Both an intimate body part, but also an intimate moment between the child and me.

### Breastfeeding in an incomprehensible reality

The experience of IPV during breastfeeding could be interpreted as breastfeeding in an incomprehensible reality. The incomprehensibility created by a partner who constantly alters his behaviour and (breastfeeding) opinions means being forced to navigate in a bizarre reality in which logic is disrupted:

He doesn’t really have a position, it’s just about opposing//When I wanted him to take her when she didn’t need to breastfeed, then I was an egoist who didn’t [breastfeed], and when I wanted to breastfeed, then that was wrong…

This incomprehensible reality brings with it a lack of opportunity to control or reflect on the situation and its breastfeeding consequences. *“Not having a voice”* to talk about IPV or not “*getting hold of”* it due to suppressed emotions results in an invisible exposedness and difficulty in genuinely existing as a human being. The presence of, and reflection with other people have the potential to protect against the incomprehensible reality of IPV. However, there is a risk that other people’s failure to adequately interpret partner behaviour could invalidate the woman’s experience:

He was praised at the maternity ward that he had been such a fantastic support. And then we come home, and I just feel like it’s the complete opposite. No one sees what’s going on.

Breastfeeding in an incomprehensible reality seems incompatible with self-image as it implies an unrecognizable weakness, as one woman describes:

It’s still some kind of… question of identity, which I struggle with, that I am the type of woman who has been subjected to violence.

This incomprehensible reality could be a reason for building façades as protection from scrutiny and consequences. Finding arguments that the partner is not a typical woman abuser or pretending to live a happy family life means reduced shame. Thus, breastfeeding in an incomprehensible reality could mean taking part in IPV concealment or even taking the blame:

Although I probably might have… understood that it is not me who should take the shame, you take it on yourself anyway. For him too. Because then I am shameful having chosen a bad father.

### Main interpretation: being forced into an existential exile that entails an ambiguous passive-active resistance

Based on a comprehensive understanding of the interpretations, IPV during the breastfeeding period could be explained and understood as *Being forced into an existential exile that entails an ambiguous passive-active resistance*. An exile occurs when a person is forced, driven, or banished from home or country. In this study, exile is understood as an existential exile from the safe and “homelike”, a homelessness in breastfeeding. The inability to authentically exist on one’s own terms as a breastfeeding mother, woman, and human being due to objectification, oppression, and persecution means an un-safe and unfree existence. This situation implies a risk of internalizing objectification and devaluation, potentially leading to a sense of not owning one’s being, body, voice, choices, feelings, and thoughts. Existential exile evokes an existential crisis due to the experience of losing the genuine self in an incomprehensible breastfeeding situation. Forced existential exile during the breastfeeding period involves a lost sense of safety and belonging and implies a deep loneliness because other people are often unaware of both the exile and its reasons.

Constantly striving to resist the existential exile to return “home” to the authentic self entails an intertwining of passive and active resistance. Passive-active resistance constitutes an ambiguity, in which passivity and activity coexist interdependently. Slowly adapting to an incomprehensible breastfeeding existence may be viewed as a passive act but are simultaneously an active act of negotiating oneself away to protect oneself and the child from exposure to IPV. Unconsciously or consciously denying exposure to IPV by passively identifying as someone without this experience coexists with constant preparedness to fend off attacks or even actively fight back. Thus, passivity encompasses activity. Seemingly active resistance and choices are also inhibited by the situation in which they exist. Being unable to recognize this ambiguity and limited freedom may lead to homelessness and an intensified experience of existential exile.

## Discussion

### Theoretical and philosophical reflections of the results

This section will focus on reflection of the main interpretation in the context of previous research, caring science, and existential philosophy to deepen the understanding of the meaning of living in existential exile due to exposure to IPV during the breastfeeding period. The results show that IPV during breastfeeding means breastfeeding under attack in an incomprehensible reality, while being objectified, abandoned, and entrapped. Women are forced into an existential exile that entails an ambiguous passive-active resistance. To our knowledge, there is a lack of recent research on women’s lived experience of IPV during breastfeeding. However, somewhat in contrast to the current study, IPV has been shown to complicate breastfeeding by contributing to negative body image (Keeling, 2012). In accordance with our study, IPV in the childbearing context has been explained as an embodied experience in an unpredictable reality (Engnes et al., 2012) wherein women are degraded, powerless, and lonely. Responsibility for the child may lead to both adaptation and resistance (Engnes et al., 2012; H. Finnbogadóttir et al., 2014; H. R. Finnbogadóttir et al., 2024; Fogarty et al., 2019).

The term *exile* has multiple meanings in different contexts (Silva Rojas et al., 2015) and may be viewed as either a forced or self-imposed exile from the “homelike” (Peters, 2008). Based on the results, it could be questioned whether a self-imposed exile is also forced, by being an active yet unfree choice imposed on the exiled through oppression and persecution. This interpretation corresponds to descriptions of exile as something always forced upon and outside the control of the exiled (Burr, 2011). Simone de Beauvoir’s existential and feministic philosophy clarifies these seemingly irreconcilable claims. She understood human existence as ambiguous, simultaneously free and unfree, situated in a certain place in time, and affected by social expectations depending on age, sex, nationality, and so on. According to de Beauvoir, social expectations place women as *the second sex* in a patriarchal world, where males are considered the norm and women the deviation. In this situation, women become objectified and defined by men and are forced into roles that are non-compliant with their own wishes, leaving them alienated from their own existence (de Beauvoir, 2024). It is reasonable to imagine that this existential exile may be further exacerbated in the context of motherhood, breastfeeding, and IPV exposure.

Being in exile imposes a feeling of homelessness, or rather a loss of “being at home” (Burr, 2011). The experience of not being at home in the current life situation is evident in the study results. Women are lost (or homeless) in their own existence due to a lack of stability and safety. They are at risk of becoming isolated from their families or other “homelike” persons due to oppression or because they are silenced and prevented from reflecting on their situation. They may be trapped in their relationships, despite no longer feeling at home. Exile works as “an othering”, making the exiled aliens, disconnected from the experience of being at home in their own existences (Peters, 2008). Not genuinely belonging to either the old or new “home” leads to an instable reality (Silva Rojas et al., 2015) that requires constant adaptation (Peters, 2008).

Living in existential exile may ambiguously evoke both a wish to re-unite with others and/or the world in the new place and a simultaneous desire to return “home” (Burr, 2011). In the context of IPV during breastfeeding, the desire to re-unite is visualized through women’s attempts to breastfeed and manage everyday tasks despite their situation, while the wish to return home is illustrated by their efforts to escape incomprehensibility through reflection with others. Other examples of resistance from the results include hiding breastfeeding from or standing firm in the decision to breastfeed in relation to one’s partner and taking responsibility for family well-being despite an incomprehensible reality. A scoping review of women’s resistance to IPV (Rajah & Osborn, 2020) highlights the importance of context and confirms the ambiguous passive-active nature of IPV resistance. It concludes that resistance could entail consciously and intentionally opposing the partner, as well as negotiating or avoiding IPV to protect oneself or preserve the harmony of the family.

The existential philosopher Martin Heidegger viewed human existence as a “being-in-the-world”. It is to be thrown into a certain situation, time, and place. The experience of homelessness and exile from one’s own being-in-the-world could therefore be viewed as a natural part of humanity. Heidegger described living according to external expectations (*das Man*) instead of one’s own existential possibilities as living unauthentically. Overcoming this existential exile and beginning to live authentically requires courage to take responsibility for one’s own being and to accept the possibilities and limitations of one’s own existence (Heidegger, 1998). To paraphrase de Beauvoir: despite the unfree situation of being a woman, the freedom to choose to live more authentically by reclaiming subjectivity and opposing oppressive norms and structures is always a possibility. This freedom is both a blessing and a burden because of the responsibility it implies (de Beauvoir, 2024). It may seem provocative to discuss freedom and responsibility in relation to IPV. It is important to emphasize that this does not agree with the women’s self-blame. IPV is part of women’s unfree situation, but the philosophical references clarify the absence of contradiction in the ambiguity of being strong and victimized, active and passive, in line with research showing that agency does not oppose victimhood (Rajah & Osborn, 2020).

In accordance with the results of this study, *exile* was recognized in a Heideggerian-inspired hermeneutic study of sexual violence. Women’s situation was interpreted as “living-in-exile”, alienated and alone (Draucker & Madsen, 1999), but the exile descriptions differed from the current study in that there was a more concrete focus on banishment from the home. One explanation for this difference may be that the life situations of the participants were more insecure than those of the participants in this study. They were also shown to dwell with violence in a world where different manifestations of violence were ever-present (Draucker & Madsen, 1999). In relation to the current study, dwelling could be interpreted as living unauthentically to protect one’s own (and the child’s) existence. Being exposed to IPV is to be forced into an unauthentic life in which objectification is internalized and one’s own self and one’s (breastfeeding) wishes have been negotiated away for the sake of safety. From a caring science perspective, inability to engage in one’s desired life projects poses a threat to health and well-being (Dahlberg, 2011). Galvin and Todres (Galvin & Todres, 2012) found that vulnerable and unfree situations impose suffering, and their descriptions of suffering are largely consistent with the current study’s results. Suffering may consist of an exile experience, through banishment, alienation, and unbelonging, but also of imprisonment, “roomlessness” (a kind of hopeless homelessness), and having no future. On an intersubjective level, suffering may consist of feeling persecuted and victimized by another person, and suffering may lead to feelings of being unable, objectified, and fragmented (Galvin & Todres, 2012). This urges healthcare professionals to be aware that a woman exposed to IPV during breastfeeding may be experiencing suffering on multiple levels, requiring caring focused on a return to authentic life. However, living in an existential exile in an incomprehensible breastfeeding existence may be an obstacle to care-seeking. In this context, it is important to bear in mind that the patients are women who are navigating an ambiguous and patriarchal world in which seemingly free and active choices and decisions may be forced, perhaps without their awareness. IPV prevention interventions has been shown to have the potential to reduce the occurrence of IPV (Alsina et al., 2023), indicating that preventive caring initiatives that enhance awareness and draw attention to the phenomenon among both individuals and society could be beneficial. However, it has also been shown that healthcare professionals struggle with a perceived lack of expertise and resources when encountering women exposed to IPV (Alshammari et al., 2023; Aregger Lundh et al., 2023). Research is needed on how care becomes caring for women experiencing IPV during breastfeeding.

### Methodological considerations

A methodological strength of this study is its applied openness and pliability towards the phenomenon throughout all research phases. The design of the participant advertisements ensured that the women’s lived experience of IPV was in focus instead of the researchers’ pre-understanding. Open and pliable interview questions further contributed to this. A limitation of the study could be that “IPV” was not mentioned in the advertisement. However, as previously mentioned, this was deemed necessary to include participants with varied IPV experiences, and it is in line with WHO recommendations to avoid “loaded” terms and to inquire about violence in multiple ways (WHO, 2016). The approach proved effective in capturing rich and diverse phenomena meanings and the lived experience of exposedness that emerged during the interviews are understood as IPV, supported by the definition described in the background.

The participant group was relatively uniform in terms of factors such as education level and heteronormativity, which is a study limitation in relation to generalization, calling for further research. In Reflective Lifeworld Research, generalization is defined as the possibility that the study results are valid outside of the actual participant group and is achieved through abstraction (Dahlberg et al., 2008). Thus, the abstraction of a main interpretation strengthens the generalizability of the study.

The conduct of interviews via encrypted video conferencing or telephone may have influenced the study, as in lifeworld research, the body is considered “lived” since it is our prerequisite for experiencing the world and, as such, is an important aspect in lifeworld interviews (Dahlberg, 2019). However, the data collection also enabled more “anonymised” participation, which may have been beneficial for this specific study. Using both written and verbal data could also be viewed as a strength of the study. Dahlberg, Dahlberg, and Nyström (Dahlberg et al., 2008) claim that written stories have the potential to reduce author impact but lack the interview’s advantage of being able to delve deeper by asking follow-up questions. They further state that saturation in data is impossible in lifeworld research, since meaning is infinite, and the focus is on variation instead of participant number. Based on the study background data, the two methods attracted diverse groups of participants, and the data contained rich variations of the phenomenon. The interview material was more detailed, while the written stories contributed with variation and enhanced the comprehension of the interviews.

## Conclusions

Being exposed to IPV during the breastfeeding period can be equated to being forced into an existential exile in which one’s own being as a breastfeeding mother is questioned because of partner oppression and persecution. Exile is an existential crisis and a lost sense of belonging that imposes a wish to “return home” to being a woman unexposed to IPV. The breastfeeding context increases women’s vulnerability to IPV due to their increased dependence on partner support. Women are forced into unauthentic lives, in which they suffer and lack the possibility to take on their desired life projects. The exile is characterized by passive-active resistance, leading to women taking responsibility for the situation despite limited freedom. Healthcare professionals need awareness of the multifaceted suffering and situatedness of women exposed to IPV during breastfeeding to prevent and reduce suffering and enhance health and well-being.

## Data Availability

Data material is stored at the University of Borås and may be made available upon reasonable request after ethical consideration.
